# The genome sequence of the greater pipefish, *Syngnathus acus *(Linnaeus, 1758)

**DOI:** 10.12688/wellcomeopenres.19528.1

**Published:** 2023-06-23

**Authors:** Kesella Scott-Somme, Sean McTierney, Rachel Brittain, Frances Perry, Mitchell Brenen, Marine Biological Association Genome Acquisition Lab, Darwin Tree of Life Barcoding collective, Wellcome Sanger Institute Tree of Life programme, Wellcome Sanger Institute Scientific Operations: DNA Pipelines collective, Tree of Life Core Informatics collective, Darwin Tree of Life Consortium

**Affiliations:** 1 The Marine Biological Association, Plymouth, England, UK

**Keywords:** Syngnathus acus, greater pipefish, genome sequence, chromosomal, Syngnathiformes

## Abstract

We present a genome assembly from an individual *Syngnathus acus* (the greater pipefish; Chordata; Actinopteri; Syngnathiformes; Syngnathidae). The genome sequence is 359.2 megabases in span. Most of the assembly is scaffolded into 22 chromosomal pseudomolecules. The mitochondrial genome has also been assembled and is 16.5 kilobases in length.

## Species taxonomy

Eukaryota; Opisthokonta; Metazoa; Eumetazoa; Bilateria; Deuterostomia; Chordata; Craniata; Vertebrata; Gnathostomata; Teleostomi; Euteleostomi; Actinopterygii; Actinopteri; Neopterygii; Teleostei; Osteoglossocephalai; Clupeocephala; Euteleosteomorpha; Neoteleostei; Eurypterygia; Ctenosquamata; Acanthomorphata; Euacanthomorphacea; Percomorphaceae; Syngnathiaria; Syngnathiformes; Syngnathoidei; Syngnathidae; Syngnathinae; *Syngnathus*, *Syngnathus acus* (Linnaeus, 1758) (NCBI:txid161584).

## Background

The greater pipefish *Syngnathus acus* is widely distributed in the north-eastern Atlantic around coastlines, to depths of 100 m. Its habitat includes macroalgae beds, intertidal rocky shores as well as sand and mud at estuarine edges ( Russel, 2002). However, it is most commonly associated with sea grass ( *Zostera* spp.) beds ( Vincent *et al.*, 1995).

*Syngnathus acus* is a demersal syngnathid, a group characterized by its tubular snout ending in a tiny mouth, and their male parental care strategies. In fish of the *Syngnathus* genus, eggs and developing embryos are enclosed within specialised brooding structures (a marsupium) located on the ventral side of male fish ( Planas, 2022). Pipefish lacking a marsupium seem to produce smaller and less developed plankton larvae, whereas those with a marsupium, like *S. acus*, give birth to fully formed juveniles which immediately resume a benthic position upon release ( Silva *et al.*, 2006).

Like most of the ray-finned fish, (Actinopterygii) fish in the Syngnathidae family feed using suction, using their specialised lengthened and fused snout to catch faster and more mobile prey ( de Lussanet & Muller, 2007).

Although ecological research on this species is lacking, the available data suggest high seasonal and spatial variability in distribution and abundances, which may be governed by temperature, habitat and prey availability and potential migratory events ( Planas, 2022). 

### Genome sequence report

**Figure 1.**
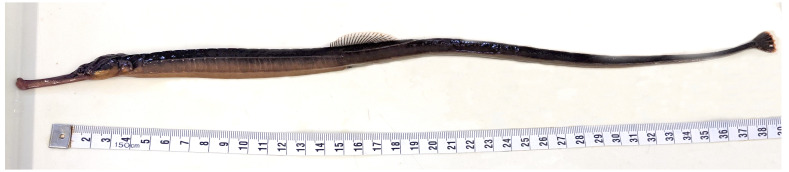
Photograph of the *Syngnathus acus* (fSynAcu2) specimen used for genome sequencing.

The genome was sequenced from one *Syngnathus acus* ( Figure 1) collected from Cawsand Bay, Plymouth, UK (50.34, –4.15). A total of 80-fold coverage in Pacific Biosciences single-molecule HiFi long reads was generated. Primary assembly contigs were scaffolded with chromosome conformation Hi-C data. Manual assembly curation corrected five missing joins or mis-joins and removed one haplotypic duplication, reducing the scaffold count by one, and decreasing the scaffold N50 by 1.84%.

**Figure 2.**
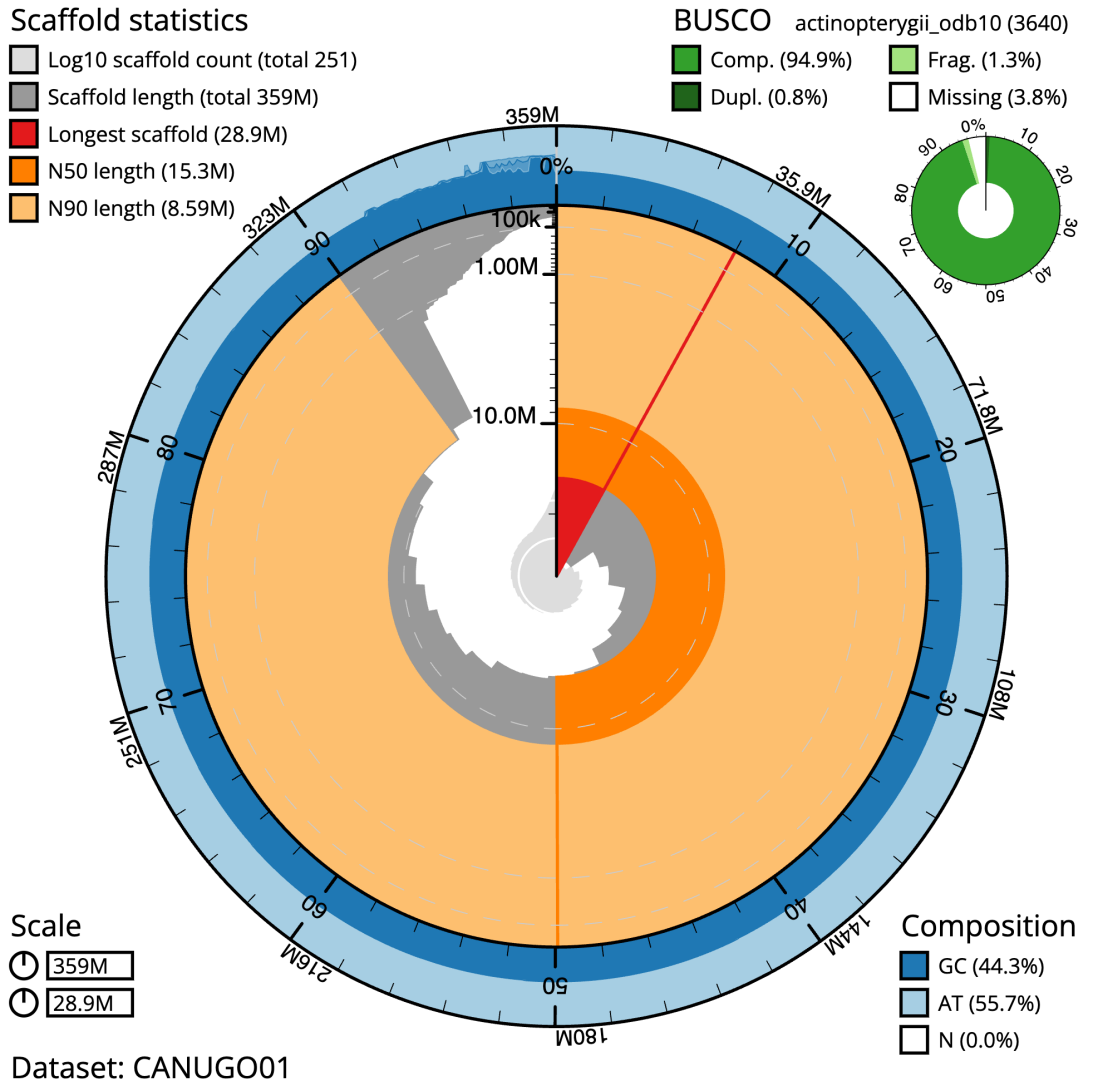
Genome assembly of *Syngnathus acus*, fSynAcu2.1: metrics. The BlobToolKit Snailplot shows N50 metrics and BUSCO gene completeness. The main plot is divided into 1,000 size-ordered bins around the circumference with each bin representing 0.1% of the 359,216,277 bp assembly. The distribution of scaffold lengths is shown in dark grey with the plot radius scaled to the longest scaffold present in the assembly (28,865,858 bp, shown in red). Orange and pale-orange arcs show the N50 and N90 scaffold lengths (15,280,236 and 8,585,000 bp), respectively. The pale grey spiral shows the cumulative scaffold e count on a log scale with white scale lines showing successive orders of magnitude. The blue and pale-blue area around the outside of the plot shows the distribution of GC, AT and N percentages in the same bins as the inner plot. A summary of complete, fragmented, duplicated and missing BUSCO genes in the actinopterygii_odb10 set is shown in the top right. An interactive version of this figure is available at https://blobtoolkit.genomehubs.org/view/fSynAcu2.1/dataset/CANUGO01/snail.

The final assembly has a total length of 359.2 Mb in 251 sequence scaffolds with a scaffold N50 of 15.3 Mb ( Table 1). Most (92.29%) of the assembly sequence was assigned to 22 chromosomal-level scaffolds. Chromosome-scale scaffolds are named by synteny based on the *S. acus* assembly GCF_901709675.1 ( Figure 2– Figure 5; Table 2). While not fully phased, the assembly deposited is of one haplotype. Contigs corresponding to the second haplotype have also been deposited. The mitochondrial genome was also assembled and can be found as a contig within the multifasta file of the genome submission.

**Table 1.** Genome data for *Syngnathus acus*, fSynAcu2.1.

Project accession data		
Assembly identifier	fSynAcu2.1	
Species	*Syngnathus acus*	
Specimen	fSynAcu2	
NCBI taxonomy ID	161584.0	
BioProject	PRJEB32743	
BioSample ID	SAMEA13854528	
Isolate information	fSynAcu2	
Assembly metrics *		*Benchmark*
Consensus quality (QV)	57.2	*≥ 50*
*k*-mer completeness	99.99%	*≥ 95%*
BUSCO **	C:94.9%[S:94.0%,D:0.8%], F:1.3%,M:3.8%,n:3,640	*C ≥ 95%*
Percentage of assembly mapped to chromosomes	92.29%	*≥ 95%*
Sex chromosomes	-	*localised homologous pairs*
Organelles	Mitochondrial genome assembled	*complete single alleles*
Raw data accessions		
PacificBiosciences SEQUEL II	ERR10677856	
Hi-C Illumina	ERR10684087	
Genome assembly		
Assembly accession	GCA_948146105.1	
*Accession of alternate haplotype*	GCA_948146095.1	
Span (Mb)	359.2	
Number of contigs	346	
Contig N50 length (Mb)	4.9	
Number of scaffolds	251	
Scaffold N50 length (Mb)	15.3	
Longest scaffold (Mb)	28.9	

* Assembly metric benchmarks are adapted from column VGP-2020 of “Table 1: Proposed standards and metrics for defining genome assembly quality” from ( Rhie *et al.*, 2021). ** BUSCO scores based on the actinopterygii_odb10 BUSCO set using v5.3.2. C = complete [S = single copy, D = duplicated], F = fragmented, M = missing, n = number of orthologues in comparison. A full set of BUSCO scores is available at https://blobtoolkit.genomehubs.org/view/fSynAcu2.1/dataset/CANUGO01/busco.

**Figure 3.**
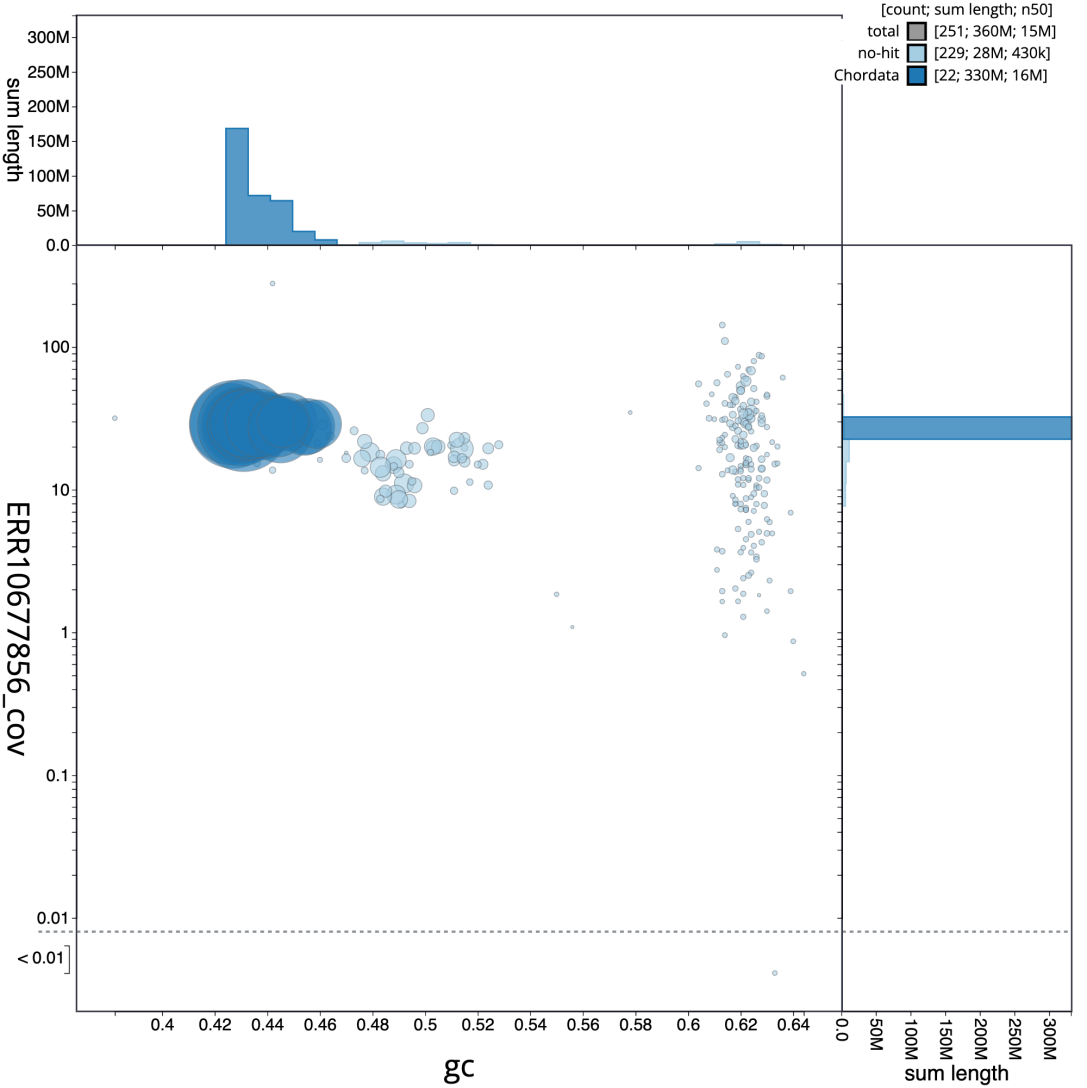
Genome assembly of *Syngnathus acus*, fSynAcu2.1: BlobToolKit GC-coverage plot. Scaffolds are coloured by phylum. Circles are sized in proportion to scaffold length. Histograms show the distribution of scaffold length sum along each axis. An interactive version of this figure is available at https://blobtoolkit.genomehubs.org/view/fSynAcu2.1/dataset/CANUGO01/blob.

**Figure 4.**
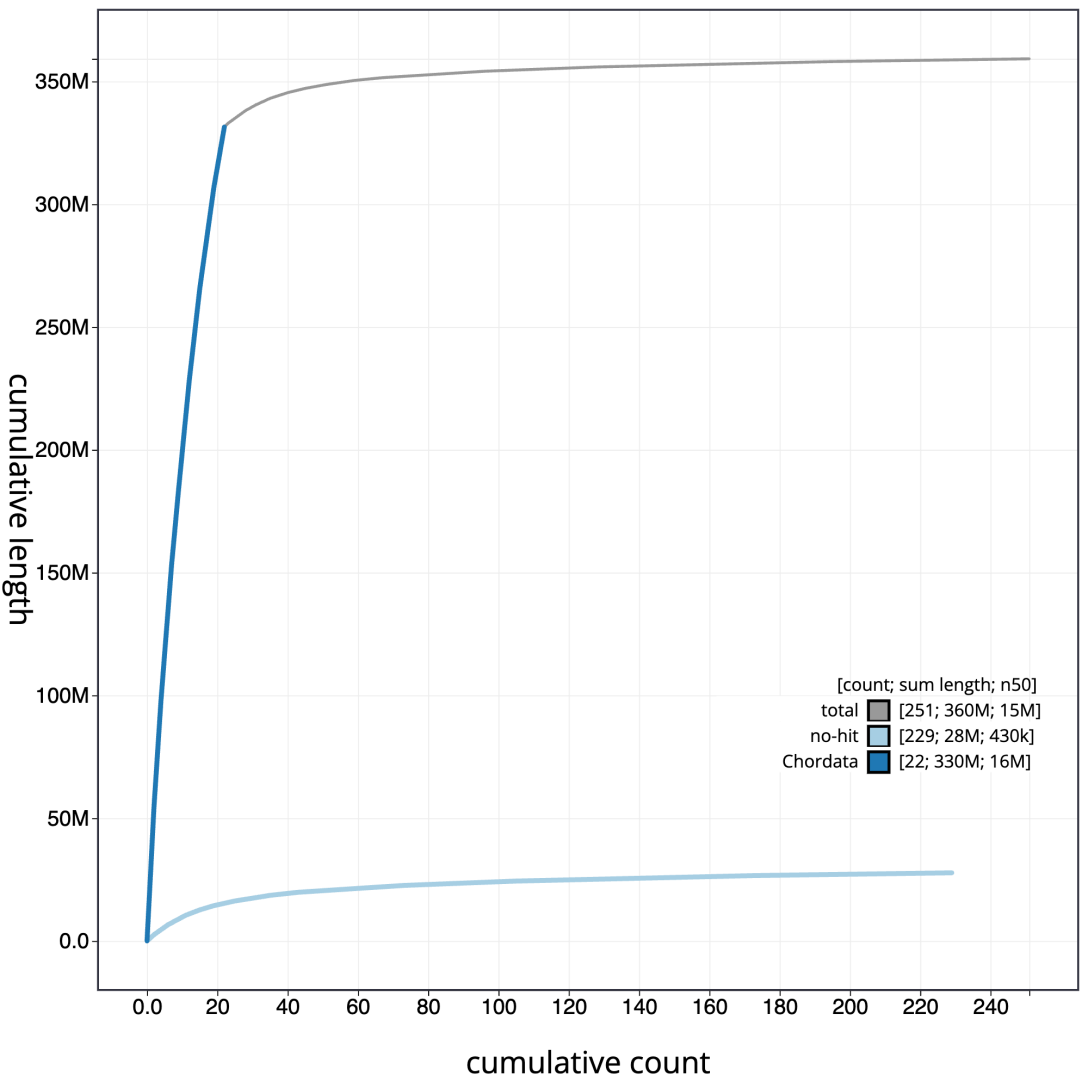
Genome assembly of *Syngnathus acus*, fSynAcu2.1: BlobToolKit cumulative sequence plot. The grey line shows cumulative length for all scaffolds. Coloured lines show cumulative lengths of scaffolds assigned to each phylum using the buscogenes taxrule. An interactive version of this figure is available at https://blobtoolkit.genomehubs.org/view/fSynAcu2.1/dataset/CANUGO01/cumulative.

**Figure 5.**
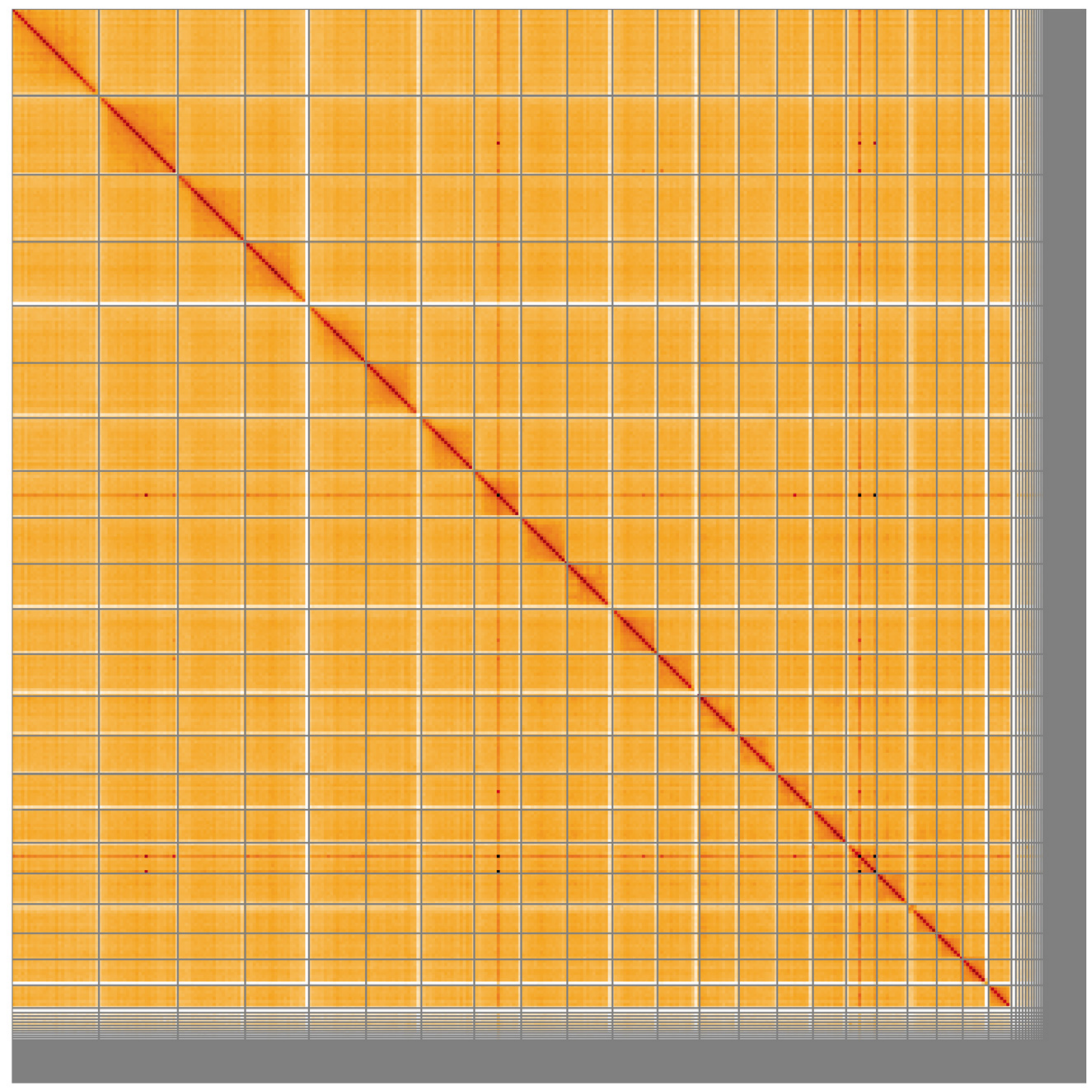
Genome assembly of *Syngnathus acus*, fSynAcu2.1: Hi-C contact map of the fSynAcu2.1 assembly, visualised using HiGlass. Chromosomes are shown in order of size from left to right and top to bottom. An interactive version of this figure may be viewed at https://genome-note-higlass.tol.sanger.ac.uk/l/?d=TVSC5z-tREy3ilKg1pCxng.

**Table 2.** Chromosomal pseudomolecules in the genome assembly of *Syngnathus acus*, fSynAcu2.

INSDC accession	Name	Length (Mb)	GC%
OX411225.1	1	18.93	43
OX411222.1	2	26.17	42.5
OX411223.1	3	22.32	42.5
OX411233.1	4	13.09	44
OX411229.1	5	15.28	43.5
OX411226.1	6	18.35	43
OX411236.1	8	11.02	45
OX411224.1	9	21.15	43
OX411221.1	10	28.87	43
OX411238.1	11	10.19	45.5
OX411234.1	12	12.68	43.5
OX411227.1	13	17.52	43
OX411237.1	14	10.14	44
OX411231.1	15	14.93	43.5
OX411230.1	16	15.01	43
OX411232.1	17	13.9	44.5
OX411239.1	19	9.71	45.5
OX411241.1	20	8.59	44.5
OX411228.1	21	15.57	43.5
OX411235.1	22	11.86	44.5
OX411242.1	23	7.6	46
OX411240.1	24	8.59	44.5
OX411243.1	MT	0.02	44.5

The estimated Quality Value (QV) of the final assembly is 57.2 with *k*-mer completeness of 99.99%, and the assembly has a BUSCO v5.3.2 completeness of 94.9% (single =94.0%, duplicated = 0.8%), using the actinopterygii_odb10 reference set ( *n* = 3,640).

Metadata for specimens, spectral estimates, sequencing runs, contaminants and pre-curation assembly statistics can be found at https://links.tol.sanger.ac.uk/species/161584.

## Methods

### Sample acquisition and nucleic acid extraction

A *Syngnathus acus* (specimen number MBA-211018-001A, individual fSynAcu2) was collected from Cawsand Bay, Plymouth, UK, from the MBA SEPIA vessel (latitude 50.34, longitude –4.15) on 2021-10-18. The specimen was captured using a Naturalist dredge and placed in a suitable container. The specimen was identified by Kesella Scott-Somme (Marine Biological Association) based on gross morphology. The fish was first anaesthetised and then overdosed using Aquased (2-phenoxyethanol). Destruction of the brain was used as a secondary method to ensure the animal was deceased before tissue sampling took place as in accordance with Schedule 1 methodology under the home office licence. The samples taken from the fish were preserved in liquid nitrogen.

DNA was extracted at the Tree of Life laboratory, Wellcome Sanger Institute (WSI). The fSynAcu2 sample was weighed and dissected on dry ice with tissue set aside for Hi-C sequencing. Somatic tissue was cryogenically disrupted to a fine powder using a Covaris cryoPREP Automated Dry Pulveriser, receiving multiple impacts. High molecular weight (HMW) DNA was extracted using the Qiagen MagAttract HMW DNA extraction kit. HMW DNA was sheared into an average fragment size of 12–20 kb in a Megaruptor 3 system with speed setting 30. Sheared DNA was purified by solid-phase reversible immobilisation using AMPure PB beads with a 1.8X ratio of beads to sample to remove the shorter fragments and concentrate the DNA sample. The concentration of the sheared and purified DNA was assessed using a Nanodrop spectrophotometer and Qubit Fluorometer and Qubit dsDNA High Sensitivity Assay kit. Fragment size distribution was evaluated by running the sample on the FemtoPulse system.

### Sequencing

Pacific Biosciences HiFi circular consensus DNA sequencing libraries were constructed according to the manufacturers’ instructions. DNA sequencing was performed by the Scientific Operations core at the WSI on the Pacific Biosciences SEQUEL II (HiFi) instruments. Hi-C data were also generated from tissue of fSynAcu2 using the Arima2 kit and sequenced on the Illumina NovaSeq 6000 instrument.

### Genome assembly, curation and evaluation

Assembly was carried out with Hifiasm ( Cheng *et al.*, 2021) and haplotypic duplication was identified and removed with purge_dups ( Guan *et al.*, 2020). The assembly was then scaffolded with Hi-C data ( Rao *et al.*, 2014) using YaHS ( Zhou *et al.*, 2023). The assembly was checked for contamination as described previously ( Howe *et al.*, 2021). Manual curation was performed using HiGlass ( Kerpedjiev *et al.*, 2018) and Pretext ( Harry, 2022). The mitochondrial genome was assembled using MitoHiFi ( Uliano-Silva *et al.*, 2022), which runs MitoFinder ( Allio *et al.*, 2020) or MITOS ( Bernt *et al.*, 2013) and uses these annotations to select the final mitochondrial contig and to ensure the general quality of the sequence.

A Hi-C map for the final assembly was produced using bwa-mem2 ( Vasimuddin *et al.*, 2019) in the Cooler file format ( Abdennur & Mirny, 2020). To assess the assembly metrics, the *k*-mer completeness and QV consensus quality values were calculated in Merqury ( Rhie *et al.*, 2020). This work was done using Nextflow ( Di Tommaso *et al.*, 2017) DSL2 pipelines “sanger-tol/readmapping” ( Surana *et al.*, 2023a) and “sanger-tol/genomenote” ( Surana *et al.*, 2023b). The genome was analysed within the BlobToolKit environment ( Challis *et al.*, 2020) and BUSCO scores ( Manni *et al.*, 2021; Simão *et al.*, 2015) were calculated.

Table 3 contains a list of relevant software tool versions and sources.

**Table 3.** Software tools: versions and sources.

Software tool	Version	Source
BlobToolKit	4.0.7	https://github.com/blobtoolkit/blobtoolkit
BUSCO	5.3.2	https://gitlab.com/ezlab/busco
Hifiasm	0.16.1-r375	https://github.com/chhylp123/hifiasm
HiGlass	1.11.6	https://github.com/higlass/higlass
Merqury	MerquryFK	https://github.com/thegenemyers/MERQURY.FK
MitoHiFi	2	https://github.com/marcelauliano/MitoHiFi
PretextView	0.2	https://github.com/wtsi-hpag/PretextView
purge_dups	1.2.3	https://github.com/dfguan/purge_dups
sanger-tol/genomenote	v1.0	https://github.com/sanger-tol/genomenote
sanger-tol/readmapping	1.1.0	https://github.com/sanger-tol/readmapping/tree/1.1.0
YaHS	yahs-1.2a	https://github.com/c-zhou/yahs

### Legal and ethical review process for DToL GAL submitted materials

The materials that have contributed to this genome note have been supplied by a Darwin Tree of Life Partner. The submission of materials by a Darwin Tree of Life Partner is subject to the **‘Darwin Tree of Life Project Sampling Code of Practice’**, which can be found in full on the Darwin Tree of Life website here. By agreeing with and signing up to the Sampling Code of Practice, the Darwin Tree of Life Partner agrees they will meet the legal and ethical requirements and standards set out within this document in respect of all samples acquired for, and supplied to, the Darwin Tree of Life Project.

Further, the Wellcome Sanger Institute employs a process whereby due diligence is carried out proportionate to the nature of the materials themselves, and the circumstances under which they have been/are to be collected and provided for use. The purpose of this is to address and mitigate any potential legal and/or ethical implications of receipt and use of the materials as part of the research project, and to ensure that in doing so we align with best practice wherever possible. The overarching areas of consideration are:

- Ethical review of provenance and sourcing of the material
- Legality of collection, transfer and use (national and international)

Each transfer of samples is further undertaken according to a Research Collaboration Agreement or Material Transfer Agreement entered into by the Darwin Tree of Life Partner, Genome Research Limited (operating as the Wellcome Sanger Institute), and in some circumstances other Darwin Tree of Life collaborators.

## Data Availability

European Nucleotide Archive: *Syngnathus acus* (greater pipefish). Accession number PRJEB32743; https://identifiers.org/ena.embl/PRJEB32743. ( Wellcome Sanger Institute, 2023) The genome sequence is released openly for reuse. The *Syngnathus acus* genome sequencing initiative is part of the Darwin Tree of Life (DToL) project. The genome will be annotated using available RNA-Seq data and presented through the Ensembl pipeline at the European Bioinformatics Institute. All raw sequence data and the assembly have been deposited in INSDC databases. Raw data and assembly accession identifiers are reported in Table 1.

## Author information

Members of the Marine Biological Association Genome Acquisition Lab are listed here: https://doi.org/10.5281/zenodo.4783604.

Members of the Darwin Tree of Life Barcoding collective are listed here: https://doi.org/10.5281/zenodo.4893703.

Members of the Wellcome Sanger Institute Tree of Life programme are listed here: https://doi.org/10.5281/zenodo.4783585.

Members of Wellcome Sanger Institute Scientific Operations: DNA Pipelines collective are listed here: https://doi.org/10.5281/zenodo.4790455.

Members of the Tree of Life Core Informatics collective are listed here: https://doi.org/10.5281/zenodo.5013541.

Members of the Darwin Tree of Life Consortium are listed here: https://doi.org/10.5281/zenodo.4783558.
