# Supplementary material for: The genome sequence of the greater pipefish, Syngnathus acus (Linnaeus, 1758)
Source: Wellcome Open Res. 2023 Jun 23;8:274. [Version 1] doi: 10.12688/wellcomeopenres.19528.1 (PMC11167339; doi:10.12688/wellcomeopenres.19528.1)
Supplement: Peer review history

# Reviewer report: Reviewer response for version 1

**DOI:** 10.21956/wellcomeopenres.21631.r84109

**Published:** 2024-06-11

**Authors:** Maria Nilsson

**Affiliations:** 1 Senckenberg Biodiversity and Climate Research Centre, Frankfurt am Main, Germany

The complete nuclear and mitochondrial genome of the greater pipefish was sequenced. The pipefish are closely related to sea horses and have evolved a highly modified body plan.

 The genome size is 359Mb. It was not possible to fully phase the assembly. The contig N50 is 4.9Mb, which indicate that the genome is of high contiguity. The complete BUSCO genes are at 94.9%

 Minor comments:

 Background: the second sentence “Its habitat includes macroalgae beds, intertidal rocky shores as well as sand and mud at estuarine edges” sounds weird. It sounds like the pipefish live in mud, which I do not think is correct.

 The mitochondrial genome has not been deposited or separated from the assembly. That makes it difficult for someone that is only interested in the MT genome. It would recommend making it available on its own. Or maybe it is already done, but this is not fully clear from the sentence “The mitochondrial genome was also assembled and can be found as a contig within the multifasta file of the genome submission”.

 conclusions: The assembly and the assembly report reads well and the high contiguity genome is a valuable addition to understanding the evolution of the highly morphologically  modified seahorses/pipefishes.

Are sufficient details of methods and materials provided to allow replication by others?

Yes

Is the rationale for creating the dataset(s) clearly described?

Yes

Are the datasets clearly presented in a useable and accessible format?

Yes

Are the protocols appropriate and is the work technically sound?

Yes

Reviewer Expertise:

phylogenomics, transposable elements, mitogenomics

I confirm that I have read this submission and believe that I have an appropriate level of expertise to confirm that it is of an acceptable scientific standard.

# Reviewer report: Reviewer response for version 1

**DOI:** 10.21956/wellcomeopenres.21631.r84108

**Published:** 2024-06-10

**Authors:** Jayan D.M Senevirathna

**Affiliations:** 1 Uva Wellassa University, Badulla, Uva Province, Sri Lanka

The manuscript is well-written in a attractive way. The data is clear and less errors.

 Suggestions:

 The Specimen should be preserved in a museum and record the sample ID of the museum sample.

 Table 2 lengths (Mb) should be indicate in three digits.

 Corrections:

 latitudes and longitudes of the sampling location should be written in the correct way.

 Part sequencing >> Hi-C data were also generated from **HMW DNA of the** tissue of fSynAcu2 .................

 Table 2 contains 24 chromosome level scaffolds, however in the text it has mentioned 22. If it is only autosomes, please mention.

Are sufficient details of methods and materials provided to allow replication by others?

Yes

Is the rationale for creating the dataset(s) clearly described?

Yes

Are the datasets clearly presented in a useable and accessible format?

Yes

Are the protocols appropriate and is the work technically sound?

Yes

Reviewer Expertise:

Genetics and Molecular Biology, Aquatic Biotechnology, Zoology

I confirm that I have read this submission and believe that I have an appropriate level of expertise to confirm that it is of an acceptable scientific standard.
